# An Insight into the Structural Diversity and Clinical Applicability of Polyurethanes in Biomedicine

**DOI:** 10.3390/polym12051197

**Published:** 2020-05-24

**Authors:** Laura-Cristina Rusu, Lavinia Cosmina Ardelean, Adriana-Andreea Jitariu, Catalin Adrian Miu, Caius Glad Streian

**Affiliations:** 1 Department of Oral Pathology, “Victor Babes” University of Medicine and Pharmacy Timisoara, 2 Eftimie Murgu sq, 300041 Timisoara, Romania; laura.rusu@umft.ro; 2 Department of Technology of Materials and Devices in Dental Medicine, “Victor Babes” University of Medicine and Pharmacy Timisoara, 2 Eftimie Murgu sq, 300041 Timisoara, Romania; 3 Department of Microscopic Morphology/Histology and Angiogenesis Research Center Timisoara, “Victor Babes” University of Medicine and Pharmacy Timisoara, 2 Eftimie Murgu sq, 300041 Timisoara, Romania; jitariu.andreea@umft.ro; 4 3rd Department of Orthopaedics-Traumatology, “Victor Babes” University of Medicine and Pharmacy Timisoara, 2 Eftimie Murgu sq, 300041 Timisoara, Romania; miu.catalin@umft.ro; 5 Department of Cardiac Surgery, “Victor Babes” University of Medicine and Pharmacy Timisoara, 2 Eftimie Murgu sq, 300041 Timisoara, Romania; streian.caius@umft.ro

**Keywords:** polyurethanes, biomedicine, carriers, vegetal extracts, surgical adhesives, cardiac valves, scaffolds, coatings, resin-based dental composites, prosthetic resins, adhesive system, electrospinning, bioadhesives

## Abstract

Due to their mechanical properties, ranging from flexible to hard materials, polyurethanes (PUs) have been widely used in many industrial and biomedical applications. PUs’ characteristics, along with their biocompatibility, make them successful biomaterials for short and medium-duration applications. The morphology of PUs includes two structural phases: hard and soft segments. Their high mechanical resistance featuresare determined by the hard segment, while the elastomeric behaviour is established by the soft segment. The most important biomedical applications of PUs include antibacterial surfaces and catheters, blood oxygenators, dialysis devices, stents, cardiac valves, vascular prostheses, bioadhesives/surgical dressings/pressure-sensitive adhesives, drug delivery systems, tissue engineering scaffolds and electrospinning, nerve generation, pacemaker lead insulation and coatings for breast implants. The diversity of polyurethane properties, due to the ease of bulk and surface modification, plays a vital role in their applications.

## 1. Introduction

PUs are primarily obtained from the petrochemical refining of coals and crude oil as raw materials [1,2], using by-products of plant material derived either from crops or their residues or from forestry biomass [3,4]. 

These materials, known under the general term of lignocellulosic biomass, are used to extract the proper raw material for PUs [3]. Lignin, one of the most sustainable raw materials used to produce polymers, widely used in paper and pulp industries, is the most frequently used natural polymer after cellulose [5], due to the fact that it is readily available in bulk quantities and inexpensive [3]. PU foams, known for their versatile mechanical properties, thermal insulation and low volumetric weight [3,6], are also produced through the re-polymer approach, based on lignin functionalization with diisocyanate, resulting in an electrophilic precursor polymer [7,8]. 

The initial stage in the manufacturing process of PUs implies either the use of a polymer, or a low-molecular-weight pre-polymer liquid, a monomer. The results of the main reactions in PU synthesis are represented by the formation of the carbamate (urethane) linkage. In 1849, Wurtz and Hoffman were the first to discover this linkage after evaluating the reaction between an isocyanate and a hydroxylated compound [9]. 

Following the accidental discovery of polyaddition for the synthesis of polyurethane out of poly-isocyanates by Otto Bayer in 1937, PUs were widely applied in the industrial field [9,10].

**Figure 1:**
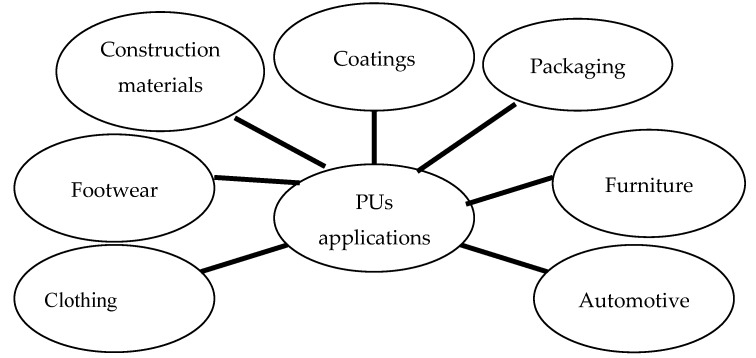
PUs’ applications.

Formed as a result of a polycondensation reaction between an isocyanate (with at least two hydroxyl groups) anda hydroxylated compound (with at least two hydroxyl groups) in the presence of a catalyst, PUs have a wide variety of industrial uses, as footwear, furniture, construction materials, automotive parts, clothing, packaging and others [1,3,9,11] (Figure 1).

Due totheir mechanical flexibility, combined with their increased tear strength, biocompatibility, biodegradability and tailorable forms, PUs have attracted the attention of biomedical device developers since 1950, when a polyester-urethane foam was first usedas a breast prosthesis coating [12]. 

In 1959, a newly developed PU foam wasapplied as a bone gap filler and immobilizing agent, followed by a polyester-urethane-based material used in the preparation of heart valves and aortic grafts [9]. Although PUs have excellent mechanical properties and chemical stability, and are easy to process, they are usually hydrophobic. Thus, they must be surface modified in order to adapt for biomedical applications.

The applications of polymers in biomedicine include orthopedics, ophthalmology, surgery, cardiology, dentistry, dialysis, and controlled delivery systems [13]. 

Polymers used in biomedicine need to meet certain requirements, such as biocompatibility, bioacceptability and biodegradability, and have to be modified either chemically or physically in order to achieve the desired properties [9]. When considering the medical field, besides their increased biocompatibility and antithrombogenic effects, PUs are also known to improve cell migration, sustain drug delivery and ensure proper organ reconstruction.

**Figure 2:**
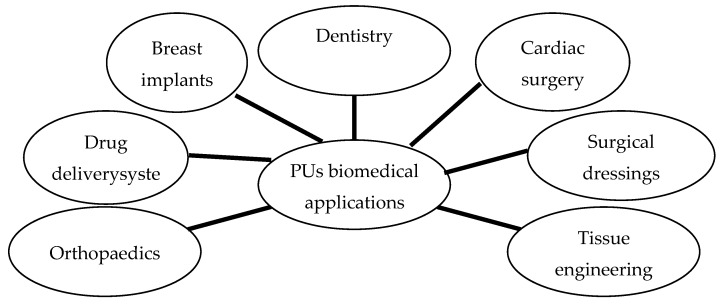
Biomedical applications of PUs.

Current biomedical application areas of PUs include antibacterial surfaces and catheters, blood oxygenators, dialysis devices, stents, cardiac valves, vascular prostheses, bioadhesives/surgical dressings/pressure-sensitive adhesives, drug delivery systems, tissue engineering scaffolds and electrospinning, nerve generation, pacemaker lead insulation and coatings for breast implants (Figure 2) [14,15,16].

Due to the multiple and extensive development of the PUs, our approach is not to be considered an exhaustive one. In this regard, the aim of this narrative review is to present the key structural properties of polyurethanes and their applications in biomedicine. In this regard, the current paper is bringing new insights to systematized knowledge in this area.

## 2. Structure and Properties

Prepared from a wide variety of materials with different properties [17], polyurethanes (PUs) are probably the most versatile group of polymeric materials. PU structures express different properties due to their ability to incorporate various functional groups such as ester and urea groups, ether, carbodiimide or aromatic rings (Table 1) in a polymeric structure [18]. 

The molecular structure of PUs may differ, from rigid crosslinked polymers (thermosettingPUs) to elastomers with linear and flexible chains (thermoplastic PUs). Thermosetting PUs contain rigid crosslinked polymers that differ from thermoplastic PUs containing linear and flexible chains [10]. 

The molecular phase of thermosetting PUs is composed of covalently linked chain networks, while that of thermoplastic PUs consists of independent, linear molecules. Unlike thermosetting PUs, thermoplastic PUs can be melted and processed by heating. Thermosetting PUs require polymerization either at room temperature or by heating and cannot be re-melted once solidified [9]. Due to their increased elastic recovery and fatigue resistance, thermoplastic PUs offer great benefits in the field of tissue engineering, particularly in applications where mechanical properties represent the main criteria of design [19]. 

Elastomeric PUs consist of soft and hard segments, containing copolymers with segmented structures composed of blocks with variable lengths. The hard segment is responsible for the increased mechanical resistance while the soft segment ensures the elastomeric behaviour of the material. Therefore, the singular molecular structure of PUs provides them with excellent properties such as elasticity, resistance to abrasion, durability, chemical stability and facile processability.

The chemical structure of the soft segments corresponds to polyols (polyethers, polyesters)while that of the hard segments corresponds to isocyanate and to the chain extender [9]. The low glass transition temperature of the soft segment provides the elastomeric properties of the material. In contrast, the hard segment possesses a high transition temperature value and a pronounced crystallinity, both associated with the mechanical strength of the material [18]. The mixture, consisting of the soft and hard segment chemistry, soft segment molecular weight, hard segment content, and degree of crystallinity, determines the mechanical properties of biodegradable PUs [20]. Direct influences on tensile strength and modulus are determined by the content and chemistry of the hard segment [21]. 

The effects of the chemical structure of the soft segment on the degradation rate are strongly related to the concentration of the labile groups and depend on hydrophilicity and crystallinity. The degree of water diffusion into the polymer is controlled by the chemical composition of the soft segment. Accordingly, the degradation rate of polyesters is directly associated with hydrophilicity [22]. An indirect relation between the increased content of the hard segment and the decreased values of the enzymatic degradation rate was reported [22]. 

Based on the chemical structure of the soft segment, polyethers are frequently used to ensure flexibility and hydrophilicity for the resorbable structure of PUs. Polyethers thus become more stable and their degradation rate is decreased [23]. Another class of soft segments is represented by the A–B–A structure of polyols, known as triblock soft segments, used in the fabrication process of PUs. Due to their versatile behaviour, triblock structures are predominately used for resorbable PUs [24].

The amounts of isocyanates and chain extenders determine the physical features of PUs. Aromatic and aliphatic isocyanates are used to fabricate biomedical PUs. In aromatic systems, isocyanates possess increased reactivity in contact withnucleophilic reagents due to their cumulative double bond sequence, consisting of nitrogen, carbon and oxygen. Although aromatic PUs possessexcellentmechanical properties, they are able to produceside effects due to their degradation, resulting in carcinogenic diamines. In consequence, aliphatic isocyanates are mostly used to reduce the potential toxicity of these materials [25]. In addition, chain extenders are represented by low-molecular-weight hydroxyl and amine-terminated compounds. Their role is to determine the morphology of the polymer. Chain extenders are composed either by diols, creating a urethane linkage [26], or by diamine, forming a urea linkage [27].

The early usage of PUs was primarily based on soft foams and non-segmented semi-crystalline fibres. Over a period of 75 years, the chemical structure of PUs showed its versatility through the isocyanate groups that react with nucleophile types and with a large variety of polyols. 

Polyols are liquids composed of two isocyanate-reacting groups that are attached to one molecule and are classified into two major categories, namely hydroxyl-terminated and amino-terminated polyols. These materials are characterized by their large variety, including polyethylene glycol, acrylic polyol, polycarbonate polyol, castor oil, polyester polyol and a mixture of these. From the polyol group, glycol includes some of the most basic structures, such as ethylene and propylene glycols; 1,6 hexanediol; 1,4 and 1,3 butanediol. The structural aspects of polyols determine the features of PUs. The molecular weight, the functionality and the hydroxyl number of the polyol chain represent the key elements of their characteristics [21,28]. 

As a result of the structural variability of PUs, their surface characteristics can be designed to serve specific applications by using modification techniques without affecting the properties of the material. The chemical structure (hydrophilicity) and the surface morphology (topography) of PUs are two essential properties that can be customized. When a PU is in a hydrophobic environment, the soft segments are preferentially segregated to the interface. If the environment is a biological fluid or blood, the hard segments mainly adsorb in the interphase. This is the reason why surface modification of PUs is an important issue in the biomedical field.In order to obtain a uniform biological response along the surface, it is of importance that, after the surface modification, the layer should be a homogeneous one. Biological or physicochemical methods, such as radiation, grafting of monomers, chemical modification, immobilization of biological molecules and silanization are applied in order to obtain surface changes [9,10,29].

Thermoplastic PU surfaces can bemodified using UV irradiation, gamma irradiation and interfacialmodification, with different materials such as polyethylene glycol, hydroxyl ethyl methacrylate, hexamethylene diamine orchitosan [30,31,32].

The physicochemical properties of PUs are able to undergo changes when in contact with other materials or with different solvent media. PUs tend to exhibit strong variations in their tensile properties, while their thermal profiles and glass transition temperature show similar values [33,34]. 

An increased number of methylene groups between hydroxyl-terminated polybutadiene (HTPB) and tetrazole moiety results in an increase in the number of hydrogen bonds. This phenomenon ensures a more effective packing of the urethane network in PUs, thus enhancing their tensile properties [33]. Moreover, it is known that solvent media based on tetrahydrofuran exerts optimizing effects on the tensile properties [33]. The calorific values of PUs are increased when changes of pristine HTPB with tetrazole derivatives occur [33]. 

PU foams represent 67% of global PU usage and are classified into flexible and rigid foams. The definition of the terms flexible and rigid, given by the American Society for Testing and Materials (ASTM), refers to “a cellular plastic [being] considered flexible if a piece eight inches by one inch can be wrapped around a one inch mandrel at room temperature”. Moreover, foams can be classified based on the stress–stain relationship. Foam structure undergoes several simultaneous processes, namely, the mixture between the reactants, the polymerisation phase and the expansion phase [35]. 

Due to their characteristics, durability and versatility, the PUfoam market is currently growing rapidly. Gas bubbles, produced during PU polymerization processes, are considered to be the key elements of the microcellular structure of PU foams [29,36].

The mechanical properties of PUs, namely the relative amounts of hard and soft segments, have important effects on the usage of these materials in different biomedical fields. This aspect is based on the role of the hard segments that act as rigid fillers, thus reinforcing the amorphous soft segment matrix, and as pseudo-net points or physical crosslinks [18].

The following polyurethanes are considered the most biocompatible and biostable: thermoplastic polyurethane elastomers; polyurethanes with siloxane segments; and nanocomposite polymers with polycarbonate soft segments and polyhedral oligomeric silsesquioxanes covalently bound to their hard segments. They are also characterized by the known excellent mechanical properties of PUs [37,38]. 

## 3. Biomedical Applications

### 3.1. Carriers for Drug Delivery Systems

Based on the final chemical version of their structure and on block building, PUs are classified into two major categories, biodegradable and bioinert. Biodegradable PUs are incorporated into drug delivery systems [39].

Carriers are used for the immobilization of biologically active substances in thepreparation of drug delivery systems. 

Usuallynatural or synthetic polymeric materials, these carriers shouldhave the right balance ofhydrophilicity/hydrophobicity, the right charge character andbiocompatibility [40,41]. 

Their form—powder, fiber, membrane, or, more recently, microparticles based on polyurethanes and microspheres obtained with poly(3-hydroxybutyrate-co-3-hydroxyvalerate)—is dependent onthe application [42].

Using PU structures as drug delivery systems in order to achieve beneficial therapeutic effects on human subjects has been proven to improve the efficiency and safety of pharmaceutical substances inserted into the human body [43].

For example, PUs containing ciprofloxacin [44,45] and norfloxacin [46] antibiotics that bear fluorine atoms can biodegrade by immersion in dimethylacetamide solutions releasing the antibiotics. The role of fluorine atoms is to augment the drugs’ bioavailability due to increased lipophilicity and cell membrane penetration.

Due to the reduced tensile strength values of PUs, recent studies have successfully applied mesoporous bioactive glass and biodegradable PUs as reservoirs for drug sustained release [47]. 

In cancer treatment, polymer nanoparticles have been investigated for their ability to improve synergistic cancer chemotherapy. Treatment strategies in malignant lesions are known to require a decrease in drug resistance and enhancement of synergistic effects. Polymer nanoparticles based on PUs can be used to co-encapsulate chemotherapeutic agents such as doxorubicin hydrochloride and doxocetal, thus achieving significant concomitant accumulation compared to free drugs [48].

In cases when PUs have been used as a drug-loading polymer in tracheal stent intubation, beneficial results have been reported, resulting in the prevention of tissue fibrosis and re-stenosis by using doxycycline (doxy) -eluting nanofibers incorporated in endotracheal stents [49]. 

PU-based tissue adhesives may also be used as delivery systems and can be engineered for slow, localized release of painkillers or antibiotics [50].

PUs are also used as carriers for vegetable extracts, such as eugenol, garlic, mistletoe, chilli pepper, and birch bark, the effects being strongly dependent upon the extract used (Table 2).

The advantages of phytocompounds incorporated in PU carriers include improved adherence to the corneal surface, reduction of inflammatory values and exceptional patient compliance [51].

Among vegetal extracts, eugenol (EU) is largely used in the chemical industry and in biomedicine. The food industry uses EU in order to improve food storage and wine preparation and to identify the chemical structure of different types of foods of vegetable and animal origin [52,53,54,55]. Natural EU is a phenylpropene extracted from the clove plant and has multiple advantages, such as facile accessibility, a long history of human consumption and a low cost [56]. In the biomedical field, EU seems to be a useful tool in both malignant lesions and infections [57]. EU is known to possess biomedical properties, such as anti-inflammatory and antimicrobial effects, as well as potential anticancer activity, and is characterized by the presence of special functional groups that ensure its particular usage in the field of biomedicine [51]. Thus, EU-modified MQ silicone resins show enhanced antibacterial properties [58] and plant extracts containing EU were associated with anti-oxidative and anti-inflammatory effects [59]. Moreover, in-vitro studies have demonstrated the beneficial antiviral effects of natural EU. EU may act as an adjuvant agent that is able to enhance the effects of different chemotherapeutics, thus increasing the efficacy of anticancer strategy treatments. In-vitro studies show that the combination of EU with cisplatin determines a synergistic inhibition of cell growth and survival and the destruction of resistant cancer stem cells [60]. 

Survivin, a protein overexpressed in both breast cancer and malignant melanomas, is effectively inhibited by EU. In malignant melanomas, more efficient anticancer effects are obtained when anti-melanoma agents such as dacarbazine are associated with EU. This combination determines an increase in the number of late apoptotic cells and is associated with low malignant cell proliferation and migration, thus becoming a potent therapeutic strategy against metastases [61]. Moreover, through the repression of gene expressions, EU inhibits the development and growth of non-small-cell lung cancer. In-vivo studies have demonstrated the inhibition of xenograft tumour progression and prolongation of overall survival rate in different histopathological types of lung cancer, thus transforming EU into a potent tumour-suppressing agent [61,62].

EU is widely used in dentistry as an antiseptic and anti-inflammatory compound. In oral therapies, the transmembrane delivery of EU is based on PU drug delivery systems [63]. Previous studiesconducted in our university [63], regarding the evaluation of encapsulation efficiency, based on UV–Vis absorption of free drug related to the quantity of EU added to synthesis, revealed a 67% active agent entrapped inside the PU structures. The bioevaluation of PU carriers used for EU showed that they are safe to use in oral therapy [63]. Another study conducted in our university assessed the effects of EU incorporated in PU structures on mitochondrial and metabolic parameters on SCC-4 human tongue squamous cell carcinoma cell line [57]. This in-vitro study concluded that EU incorporated in PU structures blocked the inhibitory effects on mitochondrial respiration [57].

PU-structure-based drug delivery systems, containing phytocompounds obtained from garlic (*Allium sativum*) and mistletoe (*Viscum album*), were also subject to testing as possible remedies for choroidal melanoma, in studies conducted in our university [64]. Hydrolysed mistletoe extract and hydrolysed garlic extract, resultingfrom the extraction protocol of phytocompounds from vegetal materials (leaves of *Viscumalbum* and *Allium sativum*), were used for the fabrication of PU structures followinga multilevel process based on the reaction betweendiisocyanates and a mixture of diols and polyols, incorporated in a spontaneous emulsification [63,65,66,67]. The long-term stability of particles toward their tendency to form aggregates of increasing size, a phenomenon known as flocculation and/or coagulation [67], was studied, and results show a higher mobility in case of solutions with PUs containing garlic, as well as mistletoe, compared tosaline solutions with empty polymer structures [64]. The efficacy of phytocompound encapsulation was also demonstrated due to the lack of active vegetable compound extracts in the polymer saline solution, confirming the literature data. The solutions containing PU structure incorporating garlic and mistletoe extracts present optimal cell viability. The results for garlic extracts in saline solution showed efficient antiproliferative effects. Due to the slow degradation rate of the polyurethane structure, the samples did not reveal any antiproliferative activity. Thus, PU structures incorporating garlic extracts can either be used in extended treatments or to improve the activity of drugs with continuous release [64]. Drug delivery systems ensure drug transportation following a long period of time [68]. After 24-h maintenance at low doses, the garlic extract exhibits antiproliferative effects, while increased dosage induced apoptosis. 

PU structures were tested as transmembranetransportsfor chili pepper extract, based on the physical and chemical features of these structures, evaluating the delivery rate [69]. Red chili pepper can be used asa remedy in different medical cases, and theirwell-known possible side effects can be easily managed using PU structures. Literature data shows that the side effects associated with chili pepper treatment are reduced following their administration as capsaicinoids (CAPs). CAPs are found in chili pepper extracts, and, due to their physiological features, they have important implications in lipolysis. In order to properly obtain CAPs from chili pepper, the literature provides significant information regarding chili pepper processing [69,70,71].

Birch bark extracts encapsulated within PU microstructures were also tested [72]. These extracts were based on the association of pure terpenoids and their esters with fatty acids (betulin and lupeol), hydrocarbons and their epoxides, etheric oils, steroids (beta-sitosterol), flavonoids (kaempferol, quercetin, naringenin) tannins, and hydroxycoumarins (umbelliferone, esculetin). Results showed an increased size in case of PU structures containing betulin or birch bark extracts [72]. These findings are associated with the presence of two functional groups in the betulin structure that play the role of chain extenders within the polymer. In order to evaluate the clinical application of the incorporated vegetal extracts in PU structures, different parameters, such as skin irritation, transepidermal water loss, erythema and skin hydration, were evaluated. However, no significant changes were found, suggesting that PU microstructures are safe products for human usage [72].

Therefore, PU structures represent excellent materials that can be used as drug delivery systems for herbal extracts [68].

### 3.2. Scaffolds

**Figure 3:**
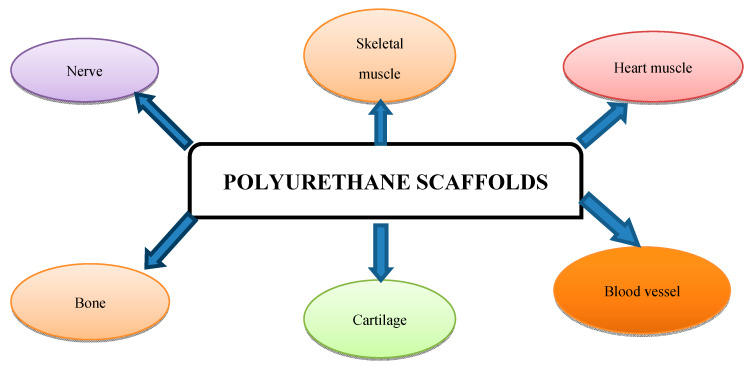
Types of PU scaffolds.

Biomedical scaffolds that are able to mimic natural tissue structures represent one of the major focuses in the field of tissue engineering (Figure 3) [73]. 

Due to their versatility, PUs can be designed to fit the requirements imposed by their final applications. The choice of their building blocks (which are used in synthesis as macrodiols, diisocyanates, and chain extenders) can be implemented to obtain biomimetic constructs, which can mimic the native tissue in terms of mechanical, morphological and surface properties [74]. In hard tissue engineering, elastomeric PUs avoid shear forces at the interface between the bone and the implant, supporting the proliferation of osteogenic cells. Soft tissues can be engineered equally efficiently, resulting in the fabrication of muscle constructs (including heart, blood vessels, cartilage and peripheral nerve regeneration) [74] (Figure 3).

Poly(carbonate-urea) urethane-based scaffolds improve cell viability and surface adherence, thus becoming potential polymer surfaces for the improvement of laryngeal reconstruction and regeneration [75,76]. 

Novel scaffolds based on PUs comprising megni oil are currently regarded as potential tools in the field of tissue engineering due to their increased antithrombogenicity [77]. 

In the field of regenerative medicine, it appears that caffeic acid phenethyl ester (CAPE) possesses inhibitory effects on cell proliferation in different cancer models. CAPE has been shown to stimulate wound re-epithelization and keratinocyte proliferation and increase the thickness of the wound epidermis [78]. 

Considering the fact that angiogenesis is currently a high-interest topic, the biomaterials industry is focused on fabricating biocompatible materials that are able to either enhance or inhibit this process. Thus, colloidal gels and aggregates based on mediated aggregation of cationic PU particles are being used to produce controlled morphological matrices [79,80,81]. Endothelial cells interconnect with the stranded colloidal network and form clusters around compact colloidal aggregates [79]. Moreover, in colloidal gels, endothelial cells form capillary-like structures, thus supporting the spatial guidance and the capacity of these materials to regulate cellular morphogenesis [79].

Bone tissue engineering has led toa series of benefits from the usage of PUs in recent years. Bone tissue regeneration and repair based on osteogenic proliferation and differentiation have been established via PU scaffolds [82]. 

PU nanofibers added with ylang ylang and zinc nitrate seem to improve calcium deposition in fabricated composites. These developed composites may serve as efficient bone tissue engineering materials as they possess excellent physical-chemical properties and biocompatibility [83]. The performance of electrospun membranes of PU/silk fibroin was also evaluated in mandibular defects. The biological efficacy of these membranes was tested regarding cell adhesion, cell proliferation and viability, calcium content and alkaline phosphatise activity. Thus, membranes associated with thin PU/silk fibroin seem to be useful biomaterials for guided bone tissue regeneration [84]. Regeneration of large bone tissue lesions requires alternative strategies based on the usage of porous scaffolds, stem cells, cytokines and different growth factors. These biomedical elements are known to improve cell proliferation, adhesion, survival and differentiation [85,86]. 

The presence of clay nanoplates in PU scaffolds was proven to stimulate osteogenic differentiation and proliferation of cultured human-adipose-derived mesenchymal stem cells [87]. These biocompatible nanocomposite scaffolds may serve as effective matrices in bone tissue reconstruction. 

Malignant lesions of the bone tissue may benefit from novel scaffold systems with multimodal therapeutic applications and co-delivery of multiple therapeutic drugs [88]. The applications of polymers in different types of human cancer are enhanced through the use of various macroscale delivery systems, especially microneedle-based devices. It is well documented that the systemic delivery of chemotherapeutics is associated with a series of side effects, whereas the local implantation of drug delivery systems on the tumour tissue is more beneficial for anticancer therapy [89,90,91]. 

### 3.3. Cardiovascular Applications

PUs are widely used in cardiovascular applications, having played a major role in the development of devices ranging from central venous catheters to the total artificial heart, because of their physiochemical properties (high shear strength, elasticity, durability, fatigue resistance, lightweight, transparency, etc.), high biocompatibility, and acceptance or tolerance in the body during healing, allowing unrestricted usage in blood-contacting devices [92,93]. PU is also used in cardiac pacing lead structures as an insulator [94]. These can be processed by extrusion and injection molding techniques to become part of devices that feel and behave like natural tissue [95,96]. 

Thermoplastic PU is known as a medical grade polymer due to its long-term durability in implants, biostability, biocompatibility, and oxidative stability. Thermoplastic PU has high shear strength, elasticity and transparency, its pliability improving its handling characteristics. Its abrasion resistance makes it ideal for use in pacemaker leads, heart pump membranes, and stent coatings. For increased flexibility, a PU–silicone copolymer may be used [97]. Because of the typical chemical end groups, PUs also exhibit potential for bulk and surface modification with a hydrophilic and hydrophobic balance. These end-group modifications can be designed to mediate and/or enhance the acceptance and healing of the device or implant. The possible in-vivo biodegradation of thermoplastic polyurethanes is, however, uncertain, as studies report different conclusions on this fact [98].

One of the most challenging fields for medical device manufacturing is that of blood–PU interaction, the major challenges being represented by biocompatibility and haemocompatibility [18]. 

Blood compatibility is one of the major criteria which limits the use of biomaterials for cardiovascular application [92,93]. For enhanced compatibility of the cardiovascular biomaterials, different surface modification strategies have been used. Organic and inorganic coatings, biofunctionalization, and biomimetic modification are only a few examples of the methods currently used to modify the structure of a particular material [99,100]. 

In order to achieve these properties, PU surfaces undergo physical and chemical changes. It is documented that almost all physical techniques only alter the surface properties, exerting no influence on the chemical structure [101,102]. Changes in the physical surface improve the haemocompatibility of PU biomaterials byensuring a structured surface and maintaining the properties of the bulk. This technique follows two directions, either platelet adhesion or protein adsorption on structured surfaces [18]. In opposition to the bioinert properties of the PU surface, cell growth and proliferation must also be generated. This phenomenon depends on the incorporation of ligands that bind to integrins. Several studies have approached this issue by using different materials, such as fibroblasts [103], gold or platinum nanoparticles [104], nitric oxide [105] and hyaluronic acid [106].

For the application of an adequate chemical technique, the key step is to obtain a bioinert PU surface by incorporating poly(ethylene glycol) (PEG) or poly(ethylene glycol) methacrylate (PEGMA). This procedure ensures that the blood components do not recognize artificial surfaces and thus do not determine immune responses. Both PEG and PEGMA are known to be hydrophilic and haemocompatible as they avoid protein adsorption due to the formation of a hydrate layer between the material surface and the surrounding medium [107]. The application of a metal coating, using titanium oxide, titanium nitride, zirconium oxide, or diamond-like carbon, is an alternative method in case of foreign reactions between blood and Pus [108,109]. 

Due to their excellent biocompatibility and bioinertness, PUs may be successfully used as thromboresistant coatings [110], being highly effective in preventing the formation of blood clots [110].

In order to improve haemocompatibility, biodegradable chitooligosaccharide-based PUs (CPUs) with lower initial decomposition temperature and higher maximum decomposition temperature compared to pure PU films were produced [111]. Changes in the physical surface improve the haemocompatibility of PU biomaterials by ensuring a structured surface while maintaining the bulk properties. Currently, this technique follows two directions, either platelet adhesion or protein adsorption on structured surfaces, being a promising approach for achieving haemocompatible PU surfaces.After testing haemocompatibility by protein absorption and platelet adhesion, CPUs proved resistant, thus showing benefits in clinical applications [111]. 

Despite the major implications of developing haemocompatibility materials containing PUs, disadvantages, such as cost or unexpected effects that activate the complement system, must be taken into consideration [112]. 

Cardiac tissue engineering also benefits from the usage of polymers. Different types of cardiac valves, including carbon and xenografts, are currently used, but their haemocompatibility, biostability, resistance to degradation and calcification, antithrombogenicity, and long-term mechanical stability are prone to further improvement [94]. The major problems of the polymeric valves are related to both design and chemical composition in order to eliminate thrombogenicity, calcification, mechanical stress, and improve durability [113]. 

In 1982, Wisman and collaborators invented a trileaflet valve fabricated from segmented polyurethane (SPU) [114]. It is characterized by a large central flow orifice, similar to tissue valves, and it has been proved to reduce turbulence and blood trauma, also ensuring high flexure endurance, great strength and nonthrombogenic characteristics [115]. Results of in-vivo evaluation have suggested that calcification could be a limiting factor to the long-term function of polymeric valves [116]. 

Surface modifications of materials have helped to improve the thrombogenicity and calcification issue, but the durability of polymeric heart valves remains a challenge. The hemodynamic performance of the valve can be increased further by designing valves with minimal stress on the leaflets [117]. The latest design of PU valve is the stent-supported bioprosthetic heart valve, where the polymeric leaflets are mounted onto flexible stents that lead to a circular orifice during forward flow [92,118]. Available studies have shown excellent hemodynamic properties equivalent to that of a tissue heart valve and a durability comparable to that of a mechanical heart valve [119].

The biocompatibility and the overall hemodynamic function and valve durability seem to improve by increasing the capability of the synthetic surface to attract endothelial cells [120].

Looking forward, the full potential of polymeric heart valves may be realized through the integration of living cells into the valve structures [121,122].

PUs have also been investigated as a substrate in cardiac stem cell therapy, in-vitro studies being carried out on the influence of patterned PU substrates on stem-cell-derived cardiomyocyte phenotype [123]. The current trend is being represented by the endothelization of the cardiac implants and utilization of induced human pluripotent stem cells [97]. 

Besides valve structures, PUs have been also used for various other cardiovascular applications, such as pacemaker leads and ventricular-assisting devices, and can be tailored to render biodegradable systems for the tissue engineering of vascular grafts and heart valves [93,95,124]. Despite their good mechanical properties, biocompatibility and haemocompatibility, PU grafts tend to degrade during long-term in-vivo functioning, due to oxidation, creating potential problems after implantation. It has been shown that chemically coating the surface with an antioxidant has been effective in reducing oxidation [125].

Beneficial results were obtained by using vascular grafts produced with PU and glycosaminoglycans [126,127,128]. PU nanofibers are non-toxic and establish a proper environment for human umbilical cord vein endothelial cells [126]. Degradable-polar hydrophobic ionic PUs and modified non-biodegradable PU scaffolds induce anti-inflammatory effects in human monocytes and macrophages [129,130,131,132]. HHHI (polymer from a family of degradable-polar hydrophobic ionic polyurethanes) was used to fabricate multifunctional PU thin films that were able to prevent blood clotting and decrease immune response [129]. These biocompatible materials suppress fibrin and thrombin formation [129,133,134]. 

PUs’ microbial resistance is ideal for preventing infection and has the potential to reduce the risk of foreign material rejection [101,135]. 

In-dwelling catheters are known to be susceptible to microbial colonization. Moreover, experimental trials show that PU catheter tubes can successfully be used for long-term catheterization of the jugular vein as they are fully functional up to several weeks [136]. Due to the increased incidence of blood stream infections in catheterized patients, the fabrication of improved catheters is one of the most challenging issues in biomedicine. Catheter biofilm models of Staphylococcus epidermidis on PU catheters associated with daptomycin and vancomycin were used in experimental models [137]. PUs and copolymer catheters associated with antibiotics proved their efficacy in reducing the biofilm mass of Staphylococcus epidermidis and other bacterial agents [137,138,139,140]. Rifampicin, known as a potent antibiotic against Gram-positive microorganisms, and miconazole, which is an antifungal agent, introduced into PU by controlled diffusion [141,142,143], are able to prevent colonization with *S. aureus*, *S. epidermis* and enterococci. Cefadroxil, added to a PU matrix, maintains its antimicrobial activity up to 5–6 days [144].

### 3.4. Wound Dressings

Topical skin adhesives in wound-healing applications are increasingly being used for replacing sutures, because of their advantages such as rapid application, less pain and better aesthetic results [9,145,146]. The most commonly used are based either on fibrin [147] or cyanoacrylates [148], but, due to their limitations, other options are being considered, including UV-curable PUs. It is also known that PU wound dressing withadded cobalt nitrate fibres is associated with increased blood compatibility [149].

The biomedical applicability of PUs is currently being extended towards the development of self-healing biomaterials. Stretchable self-healing urethane-based biomaterials are produced using supramolecular, elastomeric polyester urethane nanocomposites of poly (1,8-octanediol citrate) and hexamethylene diisocyanate reinforced with cellulose nanocrystals [150]. These novel biomaterials have become promising in the biomedical field, as they display an optimized structure with full restoration of their mechanical properties and exert no cytotoxicity, according to in-vitro tests using human dermal fibroblasts [150], and showed low toxicity levels and fibroblast proliferation in case of PU wound dressings [151]. 

### 3.5. Dental Applications

PUs have been used for a variety of dental applications, such as resin-based composite materials, coatings, maxillofacial prosthesis, dentures and other types of dental appliances. Composite resin restoration materials, originally based on poly(methyl methacrylate), filled with quartz powders, were replaced by much modern versions, based on more complex monomers, with large molecules, capable of undergoing addition polymerization, such as bisphenol glycidyl methacrylate (Bis-GMA) or urethane dimethacrylate (UDMA) [152]. 

Bis-GMA and UDMA, havenot only been replacing PMMA as a restorative material but are also used in other dental applications, such as restorative adhesive systems, bonding orthodontic brackets, temporary fillings, bridges and crowns, and dentures [153].

UDMA is also used as a cross-linking monomer in dental adhesives, together with Bis A-GMA and triethylene glycol dimethacrylate (TEGDMA), while 4-methacryloxy-ethyl trimellitate anhydride, 4-methacryloyloxy-ethyl trimellitic acid, dipentaerythritol penta acrylate monophosphate, 2-(methacryloyloxyethyl) phenyl hydrogen phosphate, and 10-methacryloyloxydecyl dihydrogen phosphate are the most used functional monomers. 2-hydroxyethyl methacrylate (HEMA), a low-molecular-weight monomer characterized by its hydrophilic properties, is another important constituent of most adhesive systems. The cross-linking monomers provide strength to the adhesive, and have hydrophobic properties which prevent water sorption of the cured adhesive, while functional monomers are responsible for the demineralization of tooth substrates and provide a chemical bond [154]. 

PUs have also been used as coatings. PU coatings have been mostly encountered as part of a coatingsystem, used as the topcoat [155]. More recently, biocompatible polymeric coatings for metallic implants, with antibacterial features, have been attempted, using poly(cyclic carbonate)-polydimethylsiloxane, reacted by aminolysis with an organoaminosilane, thus affording the formation of an urethanic polydimethylsiloxane-based material. A hybrid coating has been obtained by performing a sol-gel process on the metallic surfaces, catalyzed by phosphotungstic acid. The tests made on these hybrid coatings have shown their hydrophobic character and, due to the presence of phosphotungstic acid, the adhesion of both Gram-positive and Gram-negative bacteria has been suppressed. In addition, the coatings presented high cytocompatibility and low cytotoxicity, making them interesting candidates as biocompatible materials [156].

PUs have often been used in the field of maxillofacial prosthetics, because of their low modulus, yet high strength and elongation, necessary for maxillofacial applications [157].

A urethane-based prosthetic resin material, indicated for fabricating complete dentures, partial dentures, flippers, temporary partials, splints, night guards, and a range of orthodontic appliances, is Eclipse (Dentsply Sirona, York, PA, USA). The Eclipse Prosthetic Resin System consists of a visible-light-cured UDMA composite in three different forms, baseplate resin, set-up resin, and contour resin [158]. 

The baseplate resin is molded directly on the master cast, the messy, time-consuming denture flasking process being thus avoided. The baseplate becomes a part of the final removable denture, the set-up resin being used for teeth mounting and the contour resin for the final design. Several studies conducted in our university assessed the fatigue properties of this material, concluding that the safe use of such a denture can be guaranteed for a five-year period [158]. 

## 4. Concluding Remarks and Future Perspectives

Due to their mechanical features, ranging from rigid to flexible, PUs are present in a variety of domains, including the biomedical field [159]. 

The morphology of polyurethane, based on two structural phases—hard and soft segments—ensures high mechanical resistance, determined by the hard segment, and elastomeric behaviour, ensured by the soft segment. Therefore, the singular molecular structure provides different properties, such as elasticity, resistance to abrasion, durability, chemical stability and facile processability [9,18].

A particular category of polymers are reactive polymers (PUs included), widely used in the chemical industry. These types of polymers are able to determine different chemical reactions at the chain levels, resulting in polymer changes [160,161]. Their preparation implies two major procedures, either performing reactions on polymer chains, or adding a monomer containing a reactive group [160].

Despite their great benefits in chemical industry and technology, polymers sometimes represent risk factors for the environment. From this point of view, microplastics are a source of marine and atmospheric pollutants as well as media for the attachment of hydrophobic organic pollutants [162,163,164,165]. It appears that PU and polyamide display the most increased ability for bisphenol A sorption. Compared to other polymers such as polyethylenes, polypropylenes, or poly(vinyl chloride), in case of both PU and polyamide, sorption is almost irreversible. Microplastics thus represent environmental risk factors, due to their role as transportation vectors for bisphenol A [162]. However, the chemical industry currently benefits from the fabrication of different polymeric structures and materials that are regarded as environment-friendly. 

Due to their extensive structure and diverse properties, PUs are considered among the most bio- and blood-compatible materials. Properties such as durability, elasticity, elastomer-like character, fatigue resistance, compliance, acceptance and tolerance in the body during healing are often also associated with PUs [18]. These materials play a major role in the development of many medical devices, ranging from catheters to total artificial hearts.Their applications include artificial organs, tissue replacement and augmentation, performance-enhancing coatings, drug delivery systems and many others [166]. Due to their usage in a wide range of domains, polymeric structures represent a major challenge considering biodegradability and biocompatibility. 

To increase biostability, novel PUs with a siloxane segment, polycarbonate polyurethanes, and nanocomposite polyurethanes were developed [167]. PUs with a siloxane segment are prone to calcification during continuous in-vivo exploitation [168]. Nanocomposite polyurethanes are free of these disadvantages and have been included into clinical trials [96,169]. On the other hand, some authors reported a low rate of patency on in-vivo testing of nanocomposite polyurethane small-caliber vascular grafts implanted into the ovine carotid artery [170].

Literature data clearly indicate the potential of PUs to complement or substitute degradable polymers, such as polyester, in the replacement of damaged tissues or organs, as well as in nanomedicine, as they show superior drug encapsulation efficiency and enhanced capability to target specific tissue compartments [74,171].

Blending allows the tailoring and modulation of the properties of selected polymers. Blends are oftenfabricated by electrospinning. Electrospinning is the most promising and simple technique for manufacturing vascular grafts of polymeric materials. 

The new generation of vascular PU grafts produced by electrospinning closely meets the requirements of an ideal prosthesis [13,172,173,174].

This method allows the diameter, composition, and porosity of nanofiber scaffolds to be controlled and multilayered vascular grafts, similar to the native vessels in their physical and biological properties, to be designed [175,176]. 

All these also contribute to graft biostability and biocompatibility, making them closer to ‘‘ideal’’ variants [177,178].

Studies show that electrospun nanocomposites treated with basil oil and titanium dioxide particles exhibit a lower cellular toxicity compared to pristine polymers [179]. Electrospun composites based on PU with added peppermint and copper sulphate used to fabricate scaffolds showed low toxicity levels and improvement of blood clotting time and seem to be more effective compared to pristine PU due to the increased cell viability [74,171]. 

Hospital infections represent a great challenge for current medicine, as they are responsible for increased morbidity and mortality worldwide. Antibacterial effects and super-hydrophobic properties are shown to be induced on the surface of thermoplastic PU sheets [180]. When using a pure PVC film, bacterial adhesion showed a significant decrease in case of *S. aureus* and *E. coli* bacteria [180]. PU and silicone with incorporated copper nanoparticles have shown antimicrobial activity against infectious agents such as *Staphylococcus aureus* and *Escherichia coli*. Incorporated polymers have proved their efficacy in reducing bacterial contamination in the case of bed rails, push plates and overbed tables [181]. 

The applications of PUs in biomedicine are continuously extending, with new research being published and demonstrating that the potentials of PUs are far from fully exploited.

## Figures and Tables

**Table 1:** Usual components of PUs [17].

Component	Type	
**Diisocyanates**	Aromatic	Toluene-2,4-diisocyanate and toluene-2,6-diisocyanate, 4,4′-methylene-bis-(phenylisocyanate)
	Alicylic	Isophoronediisocyanate, 4,4′-methylene-bis(cyclohexylisocyanate)
	Aliphatic	1,6-diisocyanatohexane
**Polyols**	Aliphatic linearpolyethers	Polyethylene oxide, polypropyleneoxide poly(tetramethylene oxide) glycol
	Aromatic polyethers	Dianole 24
	Aliphatic saturatedpolyesters	Polyadipates of ethylene glycol, diethylene glycol or propylene glycol, polycaprolactonediol
**Chain extenders**	Diols	Ethylene glycol, 1,4-butanediol
	Diamines	1,2-ethylenediamine; 1,6-hexamethylene diamine
**Catalysts**	Amine	1,4-diazabicyclo-[2,2,2]-octane
	Tin	Dibutyltindilaurate

**Table 2:** PUcarriers with vegetable extracts.

Type of PU Carrier	Action	Reference
PU with eugenol	Antiseptic Anti-inflammatory	[51,57]
	Inhibitory effects on mitochondrial respiration	
PU with Allium sativum (garlic)	Antiproliferative effect Higher mobility of the compound	[64]
PU with Viscum album (mistletoe)	Antiproliferative effect	[64]
PU with chili pepper extract	Supressing angiogenesis	[69]
